# Individual differences in cortical face selectivity predict behavioral performance in face recognition

**DOI:** 10.3389/fnhum.2014.00483

**Published:** 2014-07-02

**Authors:** Lijie Huang, Yiying Song, Jingguang Li, Zonglei Zhen, Zetian Yang, Jia Liu

**Affiliations:** ^1^State Key Laboratory of Cognitive Neuroscience and Learning and IDG/McGovern Institute for Brain Research, Beijing Normal UniversityBeijing, China; ^2^Center for Collaboration and Innovation in Brain and Learning Sciences, Beijing Normal UniversityBeijing, China; ^3^School of Psychology, Beijing Normal UniversityBeijing, China

**Keywords:** object selectivity, fusiform face area, face recognition, individual differences, functional magnetic resonance imaging

## Abstract

In functional magnetic resonance imaging studies, object selectivity is defined as a higher neural response to an object category than other object categories. Importantly, object selectivity is widely considered as a neural signature of a functionally-specialized area in processing its preferred object category in the human brain. However, the behavioral significance of the object selectivity remains unclear. In the present study, we used the individual differences approach to correlate participants' face selectivity in the face-selective regions with their behavioral performance in face recognition measured outside the scanner in a large sample of healthy adults. Face selectivity was defined as the z score of activation with the contrast of faces vs. non-face objects, and the face recognition ability was indexed as the normalized residual of the accuracy in recognizing previously-learned faces after regressing out that for non-face objects in an old/new memory task. We found that the participants with higher face selectivity in the fusiform face area (FFA) and the occipital face area (OFA), but not in the posterior part of the superior temporal sulcus (pSTS), possessed higher face recognition ability. Importantly, the association of face selectivity in the FFA and face recognition ability cannot be accounted for by FFA response to objects or behavioral performance in object recognition, suggesting that the association is domain-specific. Finally, the association is reliable, confirmed by the replication from another independent participant group. In sum, our finding provides empirical evidence on the validity of using object selectivity as a neural signature in defining object-selective regions in the human brain.

## Introduction

In neurophysiological studies, a standard criterion for neural selectivity is that the response of a neuron should be at least twice as great for the preferred stimulus category as for any other stimulus category (Tovee et al., 1993). Following this principle, functional magnetic resonance Imaging (fMRI) studies have identified several object-selective regions in human ventral visual pathway, each of which responds more highly to one object category than other object categories. These regions include the fusiform face area (FFA) responding selectively to faces (Kanwisher et al., 1997), the parahippocampal place area (PPA) responding selectively to places (Epstein and Kanwisher, 1998), the extrastriate body area (EBA) responding selectively to bodies (Downing et al., 2001), and the visual word form area (VWFA) responding selectively to visual words (Cohen et al., 2000). The object selectivity was taken as a neural signature of a functionally specialized region in processing its preferred object category. However, a fundamental question remaining unclear is whether object selectivity is indeed read out for behavioral performance on object recognition.

One of the most documented object selectivity in fMRI literature is the selective response for faces. A number of face-selective regions have been identified in human occipital-temporal cortex: most notably, the FFA which is localized in the middle fusiform gyrus, the occipital face area (OFA) localized in the inferior occipital gyri (Gauthier et al., 2000), and a region in the posterior part of the superior temporal sulcus (pSTS, Allison et al., 2000; Hoffman and Haxby, 2000). The face-selective regions typically responds more than twice as strongly for faces as for non-face objects (for review, see Kanwisher, 2000, 2003), and face selectivity is defined as the response difference between faces vs. non-face objects. Prior studies suggest a functional division of labor among the three face-selective regions, with the OFA and the FFA more involved in face recognition, whereas the pSTS more involved in processing of dynamic and social information in faces (Haxby et al., 2000; Calder and Young, 2005). The role of the OFA and FFA in face recognition is supported by three lines of evidence. First, evidence from fMRI adaptation paradigms indicates that OFA responses show sensitivity to physical changes of faces (Rotshtein et al., 2005; Fox et al., 2009) and FFA responses are sensitive to identity changes (Andrews and Ewbank, 2004; Winston et al., 2004; Rotshtein et al., 2005; Fox et al., 2009). Second, recent studies with multivariate pattern analysis (MVPA) have found distinct response patterns induced by different individual faces in the OFA and FFA (Nestor et al., 2011; Goesaert and Op de Beeck, 2013). Third, more direct evidence of face-selective regions contributing to face recognition came from neuropsychological studies showing that lesions in approximately the locations of the OFA and FFA can lead to selective impairment in face recognition (i.e., acquired prosopagnosia, AP) (Damasio et al., 1982; Sergent and Signoret, 1992; Barton et al., 2002). Yet, it remains unclear whether and how face selectivity obtained in fMRI studies contributes to behavioral performance in face recognition in normal participants. Several fMRI studies have indicated that face-selective responses in the FFA and OFA are related to trial-to-trial behavioral success of face recognition. For example, the activations in the FFA and OFA were higher in trials when participants successfully detected and identified a face than when they did not (Grill-Spector et al., 2004), and the spatial patterns of activation in the FFA and OFA were more stable among correct than incorrect trials in a face discrimination task (Zhang et al., 2012).

If the face-selective responses in the FFA and OFA indeed contribute to behavioral performance of face recognition, it should be related not only to the trial-to-trial behavior success of face recognition within individual participants, but also to the individual differences in this ability across participants. Yet the evidence regarding whether the individual differences in face selectivity is related to that in face recognition ability is ambiguous. An intuitive approach to examine this issue is to compare face selectivity in individuals with normal face recognition ability with those severely impaired in this ability in the absence of obvious lesions (i.e., developmental prosopagnosia, DP) (e.g., Kress and Daum, 2003; Behrmann and Avidan, 2005; Duchaine and Nakayama, 2006). However, the findings are mixed. Some studies found that face selectivity was either absent or weakened in the FFA of DP individuals (Hadjikhani and de Gelder, 2002; DeGutis et al., 2007; Minnebusch et al., 2009; Furl et al., 2011), whereas other studies found that face selectivity in the FFA was intact in DP (Hasson et al., 2003; Behrmann et al., 2005; Avidan and Behrmann, 2009). These contradictory results may be accounted for by several possible factors, such as the lack of statistical power (i.e., small number of DP participants tested), the heterogeneous nature of DP, and the possibility that the FFA might not be the neural substrate of DP. Another approach to address the relevance of face selectivity to individual differences in face recognition ability is to examine the correlation between these two measures. To date, only one study has used this approach and shown a positive correlation between face selectivity in the FFA and face recognition ability (Furl et al., 2011). However, the correlation was examined across both DP and normal participants. Thus, it is unknown whether the correlation was partly resulted from group difference between DP and normal participants, or whether there was a linear relationship between face selectivity and face recognition ability in normal population. Therefore, in order to overcome the limitations of previous research, here we used fMRI to examine the correlation between individuals' face selectivity in face-selective regions and their face recognition ability in a large sample of normal participants.

To do this, we first measured participants' face selectivity in the face-selective regions (i.e., the FFA, OFA, and pSTS) when they viewed faces and non-face objects in the scanner (*N* = 294). Face selectivity was calculated as the z score of activation with the contrast of faces vs. non-face objects. Then, we measured the participants' face recognition ability with an old/new memory task out of the scanner. We used a difference measure between performance with faces and performance with flowers as an index of face-specific recognition ability (FRA), which isolated processes specific to face recognition by subtracting out variances reflecting domain-general cognitive processes (e.g., general visual discrimination abilities, attention, task engagement, and decision making) (Wang et al., 2012). Third, we used individual differences approach to examine whether the magnitudes of face selectivity in the face-selective regions were associated with participants' FRA, and, if established, whether the association was specific to face processing by controlling for irrelevant factors (e.g., response for objects or behavioral performance in object recognition). Finally, to ensure sufficient statistical power and replicability (Pashler and Harris, 2012), we performed a replication of the analysis with an independent large sample of participants (*N* = 201).

## Materials and methods

### Participants

Two cohorts of college students were recruited from Beijing Normal University, Beijing, China. Cohort 1 consisted of 294 participants (age: 17–24, mean age = 20.7; 155 females), and Cohort 2 consisted of 201 participants (age: 18–23, mean age = 20.3; 123 females). Participants reported normal or corrected-to-normal vision. Participants with self-reported psychiatric and neurological disorders were excluded. Both behavioral and MRI protocols were approved by the Institutional Review Board of Beijing Normal University. Written informed consent was obtained from all participants prior to the experiment. Six participants (5 females) in Cohort 1 and one male participant in Cohort 2 did not take part in the behavioral test and consequently were excluded from further analyses.

### Stimuli

A dynamic face localizer was used in the fMRI scanning (Pitcher et al., 2011), containing colored movie clips of four object categories. Movie clips of faces were filmed on a black background, and framed close-up to reveal only the faces of 7 Caucasian children as they danced or played with toys or adults (who were out of frame). Movie clips of objects, scenes and scrambled objects were included to examine the selectivity of the FFA to faces. The objects were moving toys; the scenes were mostly pastoral scenes shot from a car window while driving slowly through leafy suburbs, along with some other videos taken while flying through canyons or walking through tunnels; and the scrambled objects were constructed by scrambling each frame of the object movie clips (for more details on the stimuli, see Pitcher et al., 2011).

### fMRI scanning

Each participant attended three runs in total, each of which lasted 3 min 18 s. Each run contained two block sets, intermixed with three 18-s rest blocks at the beginning, middle, and end of the run. Each block set consisted of four blocks with four stimulus categories, with each stimulus category presented in an 18-s block that contained six 3-s clips. The order of stimulus category blocks in each run was palindromic and was randomized across runs. During the scanning, participants were instructed to passively view movie clips containing faces, objects, scenes, or scrambled objects.

### Image acquisition

Scanning was conducted on a Siemens 3T scanner (MAGENTOM Trio, a Tim system) with a 12-channel phased-array head coil at BNU Imaging Center for Brain Research, Beijing, China. Functional images were acquired using a gradient-echo echo planar imaging sequence (30 slices, repetition time (*TR*) = 2.0 s, echo time (*TE*) = 30 ms, voxel size = 3.125 × 3.125 × 4.8 mm^3^). Slices were oriented parallel to each participant's temporal cortex covering the whole brain. In addition, a high-resolution T1 weighted MPRAGE anatomical scan was acquired for registration purposes and anatomically localizing the functional activations.

### fMRI data preprocessing

Data were analyzed using tools from the Oxford Center for Functional MRI of the Brain Software Library (FSL) (Smith et al., 2004) and in-house Python codes. A 2-stage registration was used to align functional data to Montreal Neurological Institute (MNI) standard templates. First, the functional data were aligned to structural images with a linear registration; and then the structural images were warped to MNI standard template with a non-linear approach. Functional data preprocessing included high-pass temporal filtering with a high-pass cutoff of 120 s, motion correction, and spatial smoothing using a 6-mm full-width at half-maximum (FWHM) Gaussian kernel. The voxel size of functional data was resampled to 2 × 2 × 2 mm^3^.

For the functional data of each participant, the general linear model (GLM) modeled the face, object, scene, and scrambled object stimuli as explanatory variables (EVs), convolved with a hemodynamic response function (HRF). Within the time course of each EV, the onset, and duration of every stimulus was modeled. The temporal derivative of each EV was modeled to improve the sensitivity of the model. Motion parameters were entered into the GLM as confounding variables of no interest. Statistical contrasts between pairs of different object categories were evaluated. After the first level analysis, all 3 runs from each participant were combined using a fixed-effects analysis at the second level, and the resulting images were wrapped into MNI template. Finally, the resulting contrast maps from all participants were passed forward to a random-effect group-level analysis.

### ROI identification and face selectvity calculation

Z statistic image for the contrast of faces vs. objects in group-level analysis was thresholded at *z* > 2.58 (one tailed *p* < 0.005, uncorrected) and segmented into several clusters using watershed segmentation codes developed in Python (available in the scikit-image project, http://scikit-image.org). To simplify the ROI definition for a large number of participants in our study, the ROIs for each individual were defined by projecting the ROIs obtained from the group-level analysis to each individual's brain, given that the group-level analysis provided information on the location of the ROIs by summarizing the data from all participants. The FFA was defined as the region of interest (ROI), consisting of a set of contiguous voxels that were significantly activated for faces vs. objects in the fusiform gyrus in both hemispheres (30 voxels minimum). The OFA and the pSTS were defined in the same way but localized in inferior occipital cortex and the posterior STS, respectively. Face selectivity in each ROI for each participant was calculated as the average z score from the contrast of faces vs. objects across all voxels within each ROI. Note that the face selectivity of the ROI was calculated from the same set of data that were used to define the ROI; however, this bias was unlikely to affect the brain-behavior correlation, because calculation of correlation is based on the variance, not the mean. That is, the bias may inflate the mean magnitudes of face selectivity in the ROIs for all participants, but it would not inflate the individual differences (i.e., variances) of face selectivity. For further control analysis, we also extracted the average z scores in the ROIs for faces (faces > fixation) and objects (objects > fixation).

### Behavioral test

**Figure 1:**
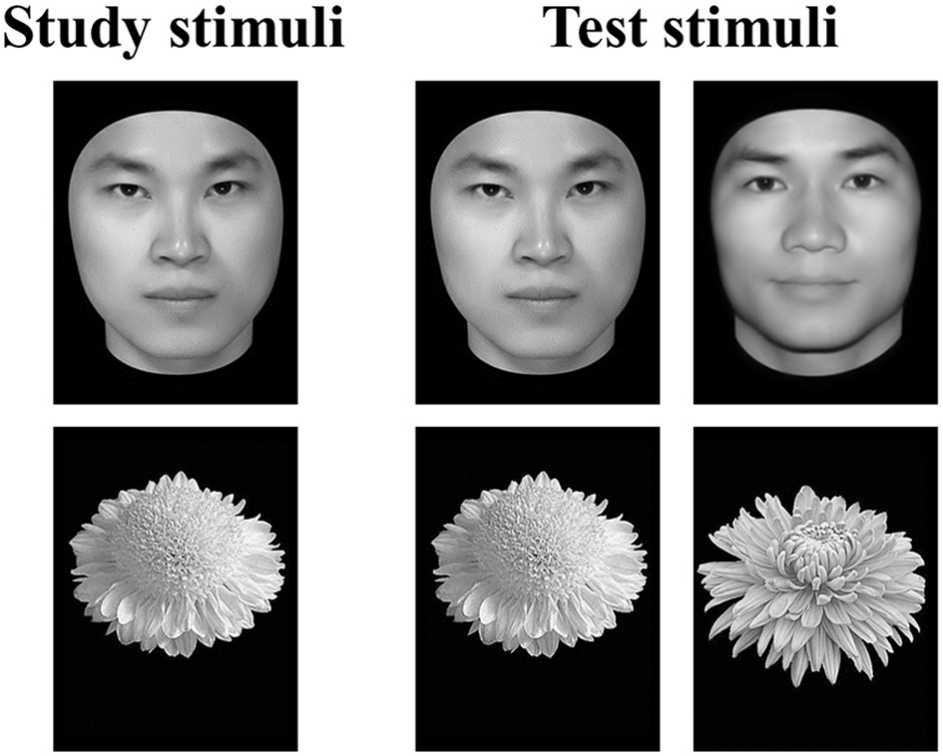
**Example stimuli and trial types in the old/new recognition task**. In the study segment, participants studied a series of images of either faces or flowers. In the test segment, the studied images were shown with new images from the same category intermixed. Participants were asked to indicate which of the images had been shown in the study segment.

The old/new recognition memory paradigm was used to measure participants' FRA. Specifically, for Cohort 1, 60 face images and 60 flower images were used (Figure 1). Face images were gray-scale adult Chinese faces with the external contours removed (leaving a roughly oval shape with no hair on the top and sides, with the addition of the neck). Flower images were gray-scale pictures of common flowers with leaves and background removed. There were two blocks in this task: a face block and a flower block, which were counterbalanced across participants. Each block consisted of one study segment and one test segment. In the study segment, 20 images of each stimuli category were shown twice. Each image was presented for 1 s with an interstimulus interval (ISI) of 0.5 s. In the test segment, the 20 studied images were shown twice, randomly intermixed with 40 new images from the same category. On presentation of each image, participants were instructed to indicate whether the image had been shown in the study segment. Cohort 2 was tested by a short version of the task (i.e., halved length), which was reported previously (Wang et al., 2012). For each stimuli category, 10 images were learned and tested (with 20 new images as distractors). Otherwise, all experimental parameters were identical to those described for Cohort 1. For each participant, a recognition score was calculated as the recognition accuracy (hits + correct rejections) for each category (face and object/flower). The FRA was calculated as the normalized residual of the face recognition score after regressing out the object (i.e., flower) recognition score.

### Voxel-wise whole-brain analysis

In addition to ROI analysis, we searched for any voxels in the whole brain that showed significant correlation between face selectivity and FRA across participants in Cohort 1. We first identified clusters of contiguous voxels showing significant correlation effect (*p* < 0.05, uncorrected), and then tested these clusters with whole brain correction (WBC) and small-volume corrections (SVC). In the WBC, the minimum cluster size above which the probability of type I error was below 0.05 was determined by the cluster program in FSL using Gaussian Random Field theory. Then, the SVCs were performed in preselected anatomical masks for regions implicated in face processing, namely, the right occipital fusiform cortex, bilateral STS, anterior temporal cortex, amygdala, OFC, and precuneus. All masks were taken from the Harvard–Oxford probabilistic structural atlas available with FSL 5.0 (FMRIB, Oxford, UK—http://www.fmrib.ox.ac.uk/fsl) with the threshold at 25%. The minimum cluster size was determined for each mask above which the probability of type I error was below 0.05.

## Results

### Face selectivity in the FFA and face recognition ability

**Figure 2:**
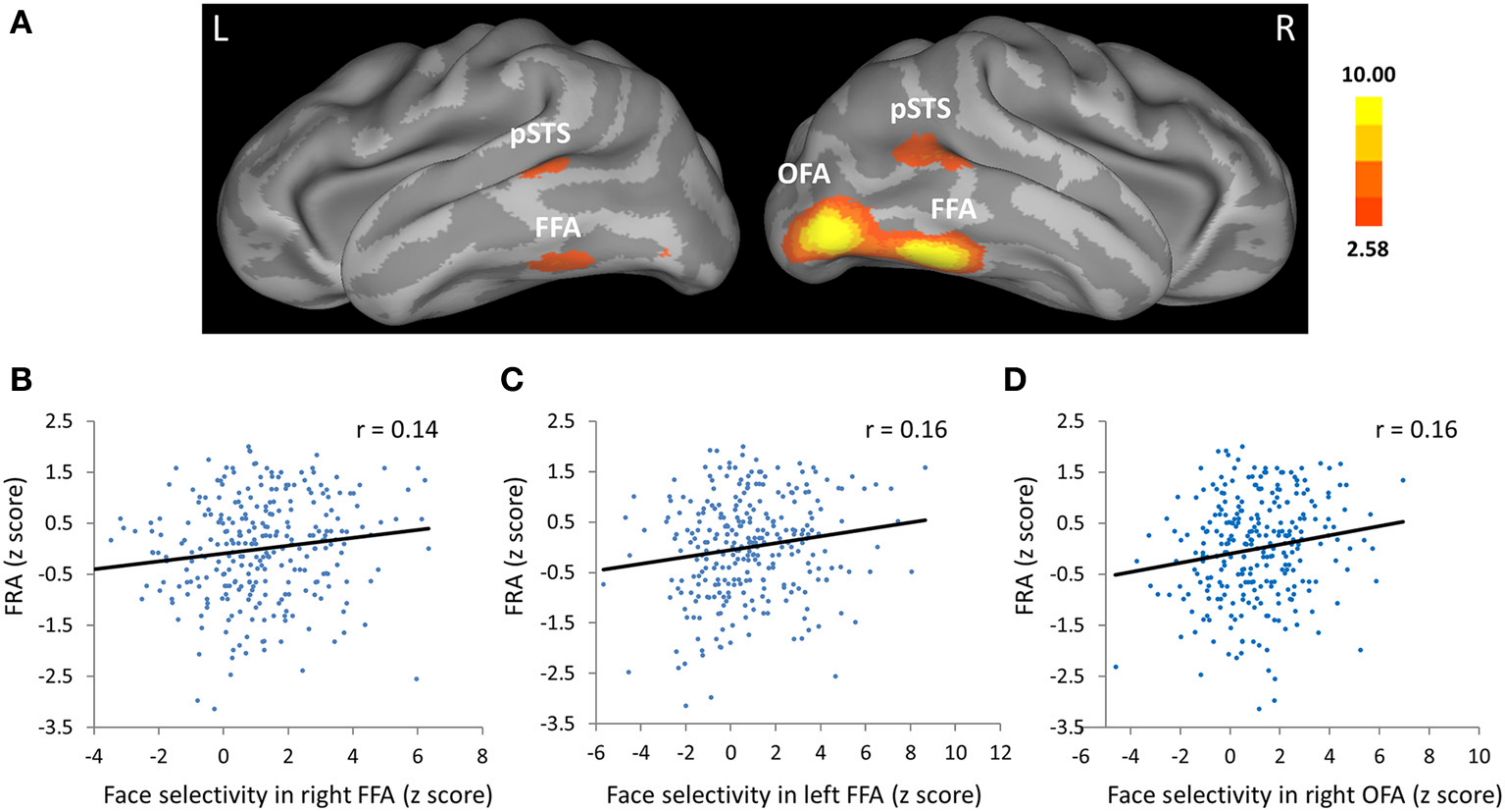
**Face selectivity in the face fusiform area (FFA) and occipital face are (OFA) was correlated with face-specific recognition ability (FRA). (A)** The FFA, OFA, and the posterior part of the superior temporal sulcus (pSTS) from group-level analysis displayed on an inflated cortical surface of MNI standard template for Cohort 1. Z statistic image for the contrast of faces vs. objects in group-level analysis was thresholded at *Z* > 2.58 (one tailed *p* < 0.005, uncorrected). **(B–D)** Scatter plots between FRA and face selectivity in the **(B)** right FFA, **(C)** left FFA, and **(D)** right OFA. The face selectivity for each participant was calculated as the average z score from the contrast of faces vs. objects across all voxels in each ROI, and the FRA was calculated as the normalized residual of the face recognition score after regressing out the object recognition score in the old/new recognition task.

Based on group-level z statistic image for the contrast of faces vs. objects (see Methods for details), the FFA was localized within the mid-fusiform gyrus in both hemispheres in two cohorts of participants (for coordinates of peak voxel and cluster size, see Table 1). Figure 2A showed the left and right FFA from the group-level analysis on an inflated cortical surface of MNI standard template. Consistent with previous literature, the right FFA was larger and more face-selective than the left FFA (see Table 1 for details).

**Table 1:** **Coordinates of peak voxels and cluster sizes of the face-selective regions from the group-level analysis**.

**Dataset**	**ROI**	**Coordinates in MNI space**			**Peak Z score**	**Cluster size**
		***x***	***y***	***z***		
Cohort 1	R FFA	42	−50	−24	12.09	700
	L FFA	−42	−50	−24	6.22	171
	R OFA	42	−92	−16	11.81	980
	R pSTS	66	−60	8	8.96	604
	L pSTS	−68	−42	4	8.19	194
Cohort 2	R FFA	42	−52	−22	12.01	603
	L FFA	−40	−52	−22	5.97	86
	R OFA	42	−82	−16	11.12	1126
	L OFA	−44	−88	−20	5.06	182
	R pSTS	64	−62	8	6.84	378
	L pSTS	−68	−52	10	4.71	43

The critical test is whether face selectivity in the FFA was correlated with the ability of face recognition. Face selectivity for each participant was calculated as the average z score from the contrast of faces vs. objects across all voxels within the ROIs, while the FRA was calculated as the normalized residual of the face recognition score after regressing out the object recognition score in the old/new recognition task (Table 2 showed descriptive statistics for this task). We found that face selectivity in the FFA of both hemispheres was positively correlated with the FRA in Cohort 1 (left FFA: Pearson's *r* = 0.16, *p* = 0.008; right FFA: Pearson's *r* = 0.14, *p* = 0.016; for scatterplots, see Figures 2B,C). Because there was no significant difference in the face selectivity-FRA correlation between the left and right FFA (Steiger's *Z*-test, *z* < 1), face selectivity in the left and right FFA was collapsed across hemispheres and used for further analyses (correlation between face selectivity of the FFA and FRA, Pearson's *r* = 0.16, *p* = 0.008). Next, we examined whether the link between face selectivity in the FFA and the FRA was specific to face processing (i.e., domain-specific), or the association was able to be accounted for by factors not specific to face processing (i.e., domain-general).

**Table 2:** **Mean Scores and standard deviations (*SD*) of the performance in the old/new recognition task and the FFA responses**.

		**Cohort 1**		**Cohort 2**	
		**Mean**	***SD***	**Mean**	***SD***
Old/new task	Face	0.74	0.09	0.78	0.09
	Flower	0.74	0.07	0.81	0.08
L FFA response	Face	5.44	2.63	6.93	3.0
	Object	4.45	2.02	5.70	2.38
	Face selectivity	0.825	2.3	1.11	2.98
R FFA response	Face	6.02	2.1	6.28	2.06
	Object	4.74	1.74	4.84	1.69
	Face selectivity	1.16	1.84	1.38	1.91

*The FFA response to faces was calculated as the average z scores across all voxels from the contrast of faces vs. fixation, and the FFA response to objects was calculated from the contrast of objects vs. fixation. Face selectivity was calculated as the average z score from the contrast of faces vs. objects*.

First, since face selectivity was calculated from the contrast of faces vs. objects, we need to rule out the possibility that the face selectivity—FRA correlation was largely resulted from a negative correlation between FFA responses to objects and FRA, rather than a positive correlation between FFA response to faces and FRA. We found that the correlation between FRA and FFA response to objects (vs. fixation) was essentially zero (Pearson's *r* = −0.003, *p* = 0.97). Further, the FRA was positively correlated with FFA response to faces, after controlling out FFA response to objects (partial *r* = 0.13, *p* = 0.03). So it is the neural response to faces, not that to objects, which led to the association between face selectivity and the FRA. Second, the face selectivity—FRA correlation was unlikely to be explained by the participants' behavioral performance on object recognition either, because there was no correlation between face selectivity and the object recognition scores (Pearson's *r* = 0, *p* = 0.99), and face selectivity was positively correlated with face recognition scores (*r* = 0.14, *p* = 0.02). Hence, the face selectivity—FRA correlation was not confounded by the variance in neural response or behavioral performance for non-face objects. Third, previous studies have shown that females are better at face recognition than males (e.g., Rehnman and Herlitz, 2007; Sommer et al., 2013), and we replicated this finding with the measure of the FRA in our study [*t*_(286)_ = 2.55, *p* = 0.01, Cohen's *d* = 0.30]. Therefore, the face selectivity—FRA association may result from the group difference between male and female participants, rather than a linear relationship across both groups of participants. To exclude this alternative, we calculated the partial correlation between face selectivity and FRA, with gender controlled out. We found that the association between FRA and face selectivity remained (partial correlation *r* = 0.14, *p* = 0.02), and thus, could not be explained by gender difference. Together, the above control analyses indicated that the association between face selectivity in the FFA and FRA is domain specific, and not able to be accounted for by the factors not specific to face processing.

Given the anatomical variability of face-selective regions across individuals, further analyses were performed to rule out the possibility that the FFA based on group-level analysis may lack specificity to tap into the FFA in individuals, especially in poor performers. First, we localized the FFAs in the poorest face recognizers (*N* = 20) at the individual level (*p* < 0.01, uncorrected), and then compared their anatomical variability with that from the best recognizer (*N* = 20). We found the mean peak voxel coordinates of the FFA in the poor group (right FFA: 42.50, −53.63, −21.75; left FFA: −40.17, −50.67, −21.50) were very close to those in the good group (right FFA: 41.50, −48.88, −22.38; left FFA: −40.71, −48.35, −23.18). Moreover, SDs of the peak voxel coordinates in the poor group (right FFA: *SDx* = 2.60, *SDy*, = 5.39, *SDz*, = 3.31 mm; left FFA: *SDx* = 3.21, *SDy*, = 7.32, *SDz*, = 4.48 mm) were comparable to those in the good group (right FFA: *SDx* = 3.35, *SDy*, = 7.14, *SDz*, = 3.33 mm; left FFA: *SDx* = 2.91, *SDy*, = 5.41, *SDz*, = 4.71 mm), indicating comparable anatomical variability of the FFA between the poor and good performers. Second, there were 9 participants fitting the definition of DP (i.e., FRA scores <2 *SD*) in Cohort 1. We recomputed the correlation between face selectivity in the FFA and FRA with the 9 participants excluded, and found the correlation remained significant (Pearson's *r* = 0.130, *p* = 0.03). Third, we defined the FFA based on group-level analysis with a more stringent threshold (one tailed *p* < 0.0001, uncorrected), and found the correlation between face selectivity in the FFA and FRA remained unchanged (Pearson's *r* = 0.15, *p* = 0.009). Taken together, these results confirmed the validity of using the group-level ROIs in the current study.

Finally, though we have revealed a face selectivity—FRA association in the FFA, the effect size of the association was rather modest (*r* = 0.16). Did this reflect the true correlation coefficient between face selectivity in the FFA and FRA, or was the observed correlation coefficient somehow biased to a low-level value? To examine the reliability of the association, we replicated this finding with another independent cohort of participants following the same procedure. The face selectivity—FRA association was confirmed in Cohort 2, and more importantly, the effect size of the association was comparable to that of Cohort 1 (Pearson's *r* = 0.15, *p* = 0.04). In addition, the association was not confounded by either the FFA response to objects (correlation between FFA response to objects and FRA, Pearson's *r* = −0.03, *p* = 0.66), or the behavioral performance on object recognition (correlation between face selectivity of FFA and the object recognition score, Pearson's *r* = −0.03, *p* = 0.72). Neither could the association be solely explained by the gender difference, because the partial correlation between face selectivity and FRA with gender controlled out was 0.12 (*p* = 0.10). In sum, although the effect size is modest, face selectivity in the FFA was reliably associated with FRA, and the association is specific to face processing.

### Face selectivity in other face-selective regions and face recognition ability

Was face selectivity in other face-selective regions associated with face recognition ability? With group-level z statistic image for the contrast of faces vs. objects, the right OFA and bilateral pSTS were obtained in Cohort 1(Figure 2A, Table 1), while the left OFA was not obtained, possibly due to large anatomical variability of the left OFA across individuals. We found that face selectivity in the right OFA was positively correlated with the FRA (Pearson's *r* = 0.16, *p* = 0.006, Figure 2D). In contrast, whereas the pSTS showed selective response for faces, its face selectivity was not correlated with the FRA (right: Pearson's *r* = −0.03, *p* = 0.59; left: Pearson's *r* = 0.06, *p* = 0.35).

These results were replicated in Cohort 2. Specifically, the bilateral OFA and pSTS were obtained in Cohort 2. Face selectivity in the OFA (right: Pearson's *r* = 0.19, *p* = 0.008; left: Pearson's *r* = 0.28, *p* < 0.001), but not that in the pSTS (right: Pearson's *r* = 0.02, *p* = 0.78; left: Pearson's *r* = −0.02, *p* = 0.78), was positively correlated with the FRA. Taken together, these results indicated that face selectivity in the FFA and OFA could predict individual differences in face recognition ability, while face selectivity in the pSTS did not link to face recognition ability, consistent with the functional division of labor among the three face-selective regions suggested in previous literature (Haxby et al., 2000; Calder and Young, 2005).

In our study, face selectivity of the ROIs was from the same dataset that was used to define the ROIs. To demonstrate that the face selectivity—FRA correlation is not subject to circularity and to further demonstrate that the results could not be accounted for by the approach of group-level ROI definition, we localized the ROIs in one cohort (i.e., Cohort 2), and then used the face selectivity in these predefined ROIs from the other cohort (i.e., Cohort 1) for the correlation analysis. The cross-cohort analysis replicated the finding from the within-cohort analysis: face selectivity in the FFA and OFA was positively correlated with the FRA in cohort 1 using the ROIs defined in cohort 2 (left FFA: Pearson's *r* = 0.15, *p* = 0.013; right FFA: Pearson's *r* = 0.14, *p* = 0.015; right OFA: Pearson's *r* = 0.15, *p* = 0.009), whereas face selectivity in the right pSTS was not correlated with the FRA (Pearson's *r* = −0.06, *p* = 0.32).

## Whole brain analysis

**Figure 3:**
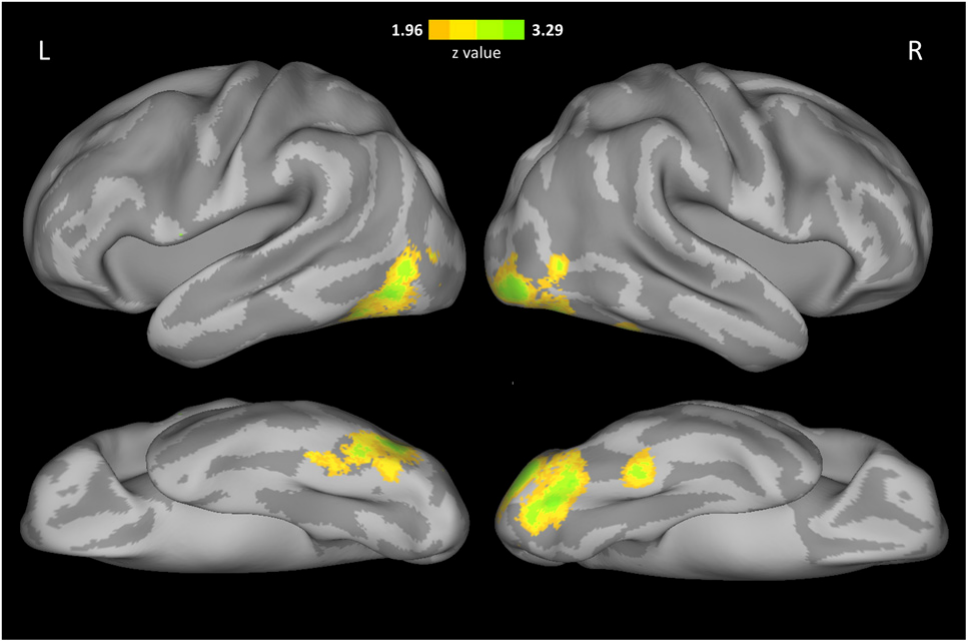
**Voxel-wise correlation between face selectivity and FRA**. The results were displayed on an inflated cortical surface of MNI standard template, thresholded at *z* > 1.96 (two tailed *p* < 0.05, uncorrected).

In addition to the ROI analysis, we searched for any voxels in the whole brain that showed correlation between face selectivity and FRA across participants in Cohort 1. The results of whole brain analysis were in agreement with those of ROI analysis (Figure 3). That is, FRA was positively correlated with face selectivity in a cluster in the right inferior occipital cortex (MNI coordinates of peak: 42, −92, −10, cluster size: 1645, peak *z*-value: 3.98), and another cluster in the left inferior occipital and fusiform cortex (MNI coordinates of peak: −42, −44, −30, cluster size: 1098, peak *z*-value: 3.95) with whole-brain correction. Then, anatomical masks were created for small volume corrections (SVC, *p* < 0.05) in regions implicated in face processing, including the right occipital fusiform cortex, the bilateral STS, anterior temporal cortex, amygdala, OFC, and precuneus. A significant positive correlation between FRA and face selectivity was found in a cluster in the right fusiform cortex (MNI coordinates of peak: 42, −44, −22, cluster size: 135, peak *z*-value: 3.03).

## Discussion

Following the criterion for neural selectivity adopted in neurophysiological research, fMRI studies have identified multiple object-selective areas in the human brain. Here in this study, we investigated the behavioral significance of object selectivity by correlating the inter-subjects variance of face selectivity in face-selective regions with individual's specific ability of recognizing faces. In two independent large samples of participants, we found that individuals with higher face selectivity in the FFA and OFA consistently exhibited better face recognition ability. Furthermore, the association of face selectivity in the FFA and face recognition ability could not be merely explained by the FFA responses to objects, general object recognition ability, or gender, suggesting that the observed association is specific to face processing. In contrast, there was no association between face selectivity in the pSTS and face recognition ability. In sum, these findings provide empirical evidence that face selectivity in the FFA and OFA contributes to behavioral performance of face recognition. The behavioral relevance of face selectivity to face recognition supports the validity of using object selectivity in defining object-selective regions, though the validity of object selectivity can also be demonstrated in other approaches.

Our study provides the first evidence that face selectivity in the FFA and OFA is related to individual differences in face recognition ability in normal population. Notably, the association remained after removing the extreme individuals fitting the definition of DP in our study. Thus, these results corroborated and extended the recent study demonstrating this association in the FFA across DP and normal participants (Furl et al., 2011). In addition, previous studies have shown that both the average response (Grill-Spector et al., 2004) and the spatial pattern of response in the FFA and OFA (Zhang et al., 2012) are involved in trial-to-trial behavioral success of recognizing faces. These two lines of evidence converged to indicate that face responses in the FFA and OFA contribute to behavioral performance of face recognition. Our results are more generally in agreement with previous studies showing that the FFA response reflects the percept of a face, rather than the physical stimuli, in binocular rivalry (Tong et al., 1998) and the Rubin vase-face illusion (Hasson et al., 2001; Andrews et al., 2002), and that the FFA responses for upright vs. inverted faces was positively correlated behavioral face-inversion effect (Yovel and Kanwisher, 2005). Taken together, these results suggest that the face-selective responses may subserve the neural correlate of face perception and face recognition. Perhaps the most convincing evidence that face-selective regions contribute to face recognition comes from the neuropsychological literature. The lesions of acquired prosopagnosic patients are usually found in ventral occipito-temporal cortex, involving both or either of the OFA and FFA, either right-sided or bilateral (Damasio et al., 1982; Sergent and Signoret, 1992; Barton et al., 2002). Importantly, results from prosopagnosic patient PS indicated that both the FFA and the OFA, and the integrity of their interaction, are necessary for successful face identification (Rossion et al., 2003; Schiltz et al., 2006; Rossion, 2008).

Further, our result suggested the association between face selectivity in the FFA and face recognition ability is domain-specific. First, the association is stimulus-specific because it is not accounted for by neural response or behavioral performance for non-face objects. Thus, a specific processing mechanism may exist for faces which distinguished from those for other object categories. Although there is alternative hypotheses proposed that the mechanisms involved in face recognition are also engaged in expert exemplar discrimination for any homogeneous visual category (Diamond and Carey, 1986; Gauthier et al., 1999, 2000), the stimulus specificity of face recognition has been supported by evidence from behavioral, developmental, electrophysiological, and clinical works, in addition to fMRI studies. Behaviorally, face recognition is more disrupted by inversion (e.g., Yin, 1969) and shows more holistic processing than object recognition (e.g., Tanaka and Farah, 1993), and there is greater development with age in face recognition than in object recognition (Carey and Diamond, 1977; Golarai et al., 2007; Weigelt et al., 2014). The neuropsychology literature of AP (Rossion et al., 2003; Busigny et al., 2010) and object agnosia (Moscovitch et al., 1997) contains evidence for a double dissociation between face and object recognition, and electrophysiological studies reveal a specialized region in monkey brain dedicated to process faces, consisting entirely of face-selective cells (e.g., Tsao et al., 2006). Interestingly, the relevance of object-selective response of an object-selective region to object recognition performance has also been demonstrated for other object categories. For example, the response to written sentences and letters strings, but not that to other object categories, in the VWFA increased linearly with reading performance (words read per minute) (Dehaene et al., 2010), and the object-selective activation in object areas (e.g., the lateral occipital complex) was positively correlated with performance of object naming across participants (Grill-Spector et al., 2000). Therefore, object selectivity may serve as a neural signature of a functionally specialized region. Note that the behavior-selectivity correlation provides sufficient but not necessary evidence to support the validity of using object selectivity to define an object-selective region.

Second, the association cannot be accounted for by domain-general cognitive processes (e.g., attention, task engagement, general visual discrimination abilities, and decision making), further suggesting the domain-specificity of the association. Although both the responses in face-selective regions (Wojciulik et al., 1998) and behavioral performance in face recognition tasks are sensitive to attention and task engagement, these general cognitive components shall be largely removed from the association after subtracting response to objects from that to faces, and subtracting object recognition scores from face recognition scores, because objects and faces likely underwent the same general cognitive processes. In addition, the correlation analysis was based on the link between in-scanner neural activation and out-of-scanner behavioral performance; therefore, those who were more attentive during scanning were not necessarily those more attentive or more engaged in the behavioral tasks out of scanner. Finally, the observation that pSTS activation did not correlate with FRA also argued against the possibility that the link between face selectivity in the FFA and face recognition ability was accounted for by general cognitive processes.

Face-selective regions are known to have anatomical variability across individuals, which may be averaged out of the group-level ROIs; however, our results were unlikely to be accounted for by the approach in defining the ROIs. First, the correlation was not resulted from the FFA variability in poor performers, because the anatomical variability of the FFA was comparable between poor and good performers, and the correlation between face selectivity in the FFA and FRA remained significant with poor performers excluded. Second, the same pattern of results was observed when the FFA was defined with a more stringent threshold or with a cross-cohort analysis, indicating that the individual-level ROI is not a critical factor to observe the behavior-selectivity correlation. Finally, the results of whole brain analysis fitted nicely with those of ROI analysis.

Comparing with previous studies, our study has a distinctive merit in methodology, that is, the association is examined in a large sample of participants, and more importantly, replicated in another independent large sample, which allows us to reveal a reliable brain-behavior association. Notably, not only the association, but also the effect size of the association was replicated in the independent sample. Yarkoni (2009) has argued that the combination of small sample sizes and stringent alpha-correction levels would lead to the grossly inflated correlations, whereas the correlations in our results are rather modest, in line with other previous studies with large sample sizes (e.g., Holmes et al., 2012; Hao et al., 2013; He et al., 2013). For the modest effect size of the association between face selectivity in the FFA and face recognition ability, there are several possible explanations. First, the responses in face-selective region as measured in our study is only one of many possible neural measures which may account for a portion of variance in face recognition ability, such as the cluster size (Furl et al., 2011) and gray matter volume of the face-selective regions (Behrmann et al., 2007; Golarai et al., 2007; Garrido et al., 2009; Dinkelacker et al., 2011), the functional connectivity (Zhu et al., 2011; Avidan et al., 2013) and anatomical connections between different face-selective regions (Thomas et al., 2009), and the connectivity between face-selective regions and the rest of the brain. Second, the old/new face recognition memory task may capture only a portion of variance in face recognition ability (Wilhelm et al., 2010; Wang et al., 2012). Third, the group-level ROIs in our study likely included some non-face-selective voxels and/or excluded some face-selective voxels in each individual, which may underestimate the true correlation coefficients between face selectivity and FRA. Therefore, further studies with face-selective ROIs defined at the individual-level may help illustrating the association more precisely. Fourth, although the dynamic localizer of Caucasian faces was sufficient to demonstrate the link between face-selective responses and face recognition ability in our study, videos of young adult Asian faces may be more desirable stimuli to tap into expert face recognition for our participants. Future research adopting optimal face stimuli may characterize the correlation more accurately. Finally, the reliability of the measurement of both face selectivity and FRA are not perfect, which may further underestimate the correlation (Schmidt and Hunter, 1999). In sum, it is not very plausible for any single neural measure to account for a large proportion of variance in a complex behavior skill such as face recognition.

In conclusion, our study provides one of the first evidence that the face selectivity in the FFA can predict face recognition ability in normal population. In our study, several issues remained unaddressed that are important topics for future research. First, the exact mechanism underlying this association is still unknown. One possibility is that higher face selectivity observed in the fMRI reflects larger number of face-responsive neurons in face-selective regions and/or shaper tuning of these neurons observed in neurophysiology studies (Tsao et al., 2006), which contribute to better behavioral performance. Another possibility is that increased face-selective response is accompanied by enhanced connectivity between different face processing regions (Saygin et al., 2012), and enhanced connectivity (e.g., more efficient transfer of face-related information) lead to better performances (e.g., Zhu et al., 2011). Future studies combining different techniques (such as single-cell recording, fMRI, and diffusion tensor imaging) are needed to explore these possibilities in depths. Second, some studies have demonstrated that FFA could be divided into two sub-regions (Pinsk et al., 2009; Weiner and Grill-Spector, 2010), and their functional roles in the association need to be further characterized. Third, although neuropsychological (Damasio et al., 1982; Sergent and Signoret, 1992; Barton et al., 2002) and transcranial magnetic stimulation (TMS) studies (Pitcher et al., 2009) have indicated the causal role of the face-selective regions in face recognition performance, we acknowledge that the correlation between face selectivity and face recognition ability in our study could be explained in the other direction. That is, for example, the FFA may be more selective to faces in good recognizers because they accumulate more information when presented with faces than poor recognizers. Finally, future studies are invited to extend the behavioral significance of object selectivity to other object categories, e.g., the place-selective response in the PPA for place recognition, and the body-selective response in the EBA for body recognition, so as to investigate whether the association between object selectivity and object recognition ability is a general principle for object recognition.

### Conflict of interest statement

The authors declare that the research was conducted in the absence of any commercial or financial relationships that could be construed as a potential conflict of interest.
